# Outperforming yet undervalued: Undergraduate women in STEM

**DOI:** 10.1371/journal.pone.0234685

**Published:** 2020-06-25

**Authors:** Brittany Bloodhart, Meena M. Balgopal, Anne Marie A. Casper, Laura B. Sample McMeeking, Emily V. Fischer

**Affiliations:** 1 Department of Psychology, California State University, San Bernardino, San Bernardino, CA, United States of America; 2 Department of Biology, Colorado State University, Fort Collins, CO, United States of America; 3 Department of Civil & Environmental Engineering, Colorado State University, Fort Collins, CO, United States of America; 4 STEM Center, Colorado State University, Fort Collins, CO, United States of America; 5 Department of Atmospheric Science, Colorado State University, Fort Collins, CO, United States of America; Hackensack University Medical Center, UNITED STATES

## Abstract

In spite of efforts to increase gender diversity across many science fields, women continue to encounter beliefs that they lack ability and talent. Undergraduate education is a critical time when peer influence may alter choice of majors and careers for women interested in science. Even in life science courses, in which women outnumber men, gender biases that emerge in peer-to-peer interactions during coursework may detract from women’s interest and progress. This is the first study of which we are aware to document that women are outperforming men in both physical and life science undergraduate courses at the same institution, while simultaneously continuing to be perceived as less-able students. This is problematic because undergraduate women may not be able to escape gender-ability stereotypes even when they are outperforming men, which has important implications for 1) the recognition of women’s achievements among their peers in undergraduate education and 2) retention of women in STEM disciplines and careers.

## Introduction

Increasing the representation of women in science, technology, engineering, and mathematics (e.g., STEM) is a mandate for U.S. higher education [1], and although women now outnumber men as college graduates, men continue to outnumber women in most STEM fields and majors [2,3]. Research has documented various barriers to women in STEM (e.g., see [4]), including pervasive gender bias (e.g. [5,6]). The negative impacts of gender bias on women’s interactions, interests, and outcomes in STEM are well-documented across the educational timeline and various sub-disciplines [7,8]. Recent experimental work has demonstrated that exposing gender bias in STEM fields produces a decreased sense of belonging and interest in participating in STEM for college women but not for college men [9]. Furthermore, students make attributions for women’s and men’s abilities in STEM when these fields are seen as unwelcoming to women. Women’s failures are attributed to internal characteristics and abilities, which ultimately lowers their sense of belonging and interest in these career fields [10].

These findings point to an important, yet under-studied, source of gender bias for women in STEM: their classmates. Student peers are likely part of the “culture” that contributes to college students’ experiences of fields and majors documented in the large swath of research on gender and STEM, but only some studies have specifically recorded gender bias among other students. For instance, other students’ explicit gender-STEM stereotypes significantly predict girls’ interests in computer science and engineering [11], while college women’s experiences of gender bias from classmates, including group-favoritism, predicts women’s aspirations to complete a STEM major or pursue a STEM career [12]. However, these studies tend to examine gender bias among student peers generally, such as gender-STEM stereotype endorsement, rather than specific bias toward girls and women in their own classrooms. One exception was conducted by Grunspan and colleagues [13], which demonstrated a pro-male bias for ratings of other students’ abilities among male students in undergraduate biology courses.

Many see women’s increased representation in fields such as life science to be examples of gains in equality (e.g., [14,15,16]). Psychological research has demonstrated that increased representation can promote better outcomes for women, particularly in STEM fields, such as a sense of belonging and interest in participation [17,18]. Furthermore, the unequal distribution of women and men in roles leads individuals to attribute stereotypic gender beliefs to individuals in those roles [19], and countries with greater representations of women in science consistently show lower gender-science stereotypes [20]. However, positive gains in representation should not necessarily be taken as a sufficient indicator of gender equality. In Grunspan and colleagues’ [13] study, undergraduate biology students continued to show gender bias by underestimating the performance of women in the class. This notably occurred in a field in which, nationally, women now outnumber men. More generally, changes in representation in particular STEM fields, such as the life sciences, are sometimes “explained away”—even by researchers—through reinforcing gender stereotypes such as women’s “relative cognitive strengths” in math compared to men’s [21].

Many studies examine the persistence of gender bias in situations where men outperform women, and this bias has been implicated as a factor contributing to their lower performance (e.g., [22,23]). However, reports show that women and girls are now catching up in many STEM fields in terms of both representation and performance [24], although these gains are underreported [25] and dismissed as being less critical measures of success (e.g., grades but not standardized test scores; [26]). As most prior work has been conducted in the context of lower performance or lower representation of women, the existing research on the impacts of gender bias is unreconciled with women’s achievement across many U.S. institutions of higher education and an examination of whether or not, and how, relative representation of women in these classes affects both men’s and women’s perceptions of women’s achievements is warranted. Thus, the purpose of the current study was to examine 1) whether college STEM students continue to hold gender biases about the abilities of their peers, particularly compared to women’s and men’s actual performance and 2) student views about the ability of their peers in life sciences versus physical sciences, where the general representation of women typically differs substantially.

## Method

Colorado State University Institutional Review Board approved this study (as Exempt 233-17H) on 10/5/2016.

### Participants

Students from a large, western U.S. university who were enrolled in nine STEM undergraduate courses were invited by their instructors to participate in a study about perceptions of classroom experiences. We recruited instructors of STEM courses that incorporate student-to-student interactions during class time (e.g., group work or laboratory work), and those who were willing to volunteer then asked their students to participate in the study. These courses were recruited because, even though the types of student interaction varied, they provided the opportunity for students to get to know one another, enabling them to identify classmates on our surveys and perhaps break down stereotyped expectations. Instructors had the option of requiring students to participate in the survey for course credit or to make it optional (either via extra credit or no credit). In all cases, students were informed that their instructor had no knowledge of their survey responses (only whether they had completed the survey), and student participants were allowed to “opt out” at the end of the survey, by indicating whether they wanted their survey responses included in the study.

There were 2,720 total students enrolled in the participating courses, including 146 students who were concurrently enrolled in two participating courses and whose grades were included for both courses. This accounted for 2,866 student grades included in the analyses. A total of 935 students participated in the survey about their perceptions of other students. Demographic information about the overall student sample and the participating students in this study are reported in Table 1. Because of the lack of power for investigating gender categories beyond men and women, these participants were not included in analyses involving the variable of participant gender.

**Table 1:** Demographic information of total student enrollment vs. participating students, by course type.

Demographics		Total Students Enrolled		Students Participating in Study	
		Life Science	Physical Science	Life Science	Physical Science
Gender					
	Women	1351	185	323	119
	Men	737	593	161	323
	Non-binary / other	n/a	n/a	1	2
Year in College					
	1^st^	1259	198	194	119
	2^nd^	423	231	128	125
	3^rd^	228	120	72	144
	4^th^ +	178	226	92	224
Ethnicity					
Minority		527	136		
	Latinx			53	39
	Asian			27	39
	Middle-Easterner			5	27
	Black			2	9
	Native American			16	10
	Other			19	13
Non-Minority		1561	642		
	White			403	352
Major					
	STEM	1537	683	381	398
	Non-STEM	551	94	105	50
Course Level					
	100	1567	445	233	239
	200	334	19	111	9
	300	155	156	120	112
	400	32	158	22	89
TOTAL		2088	778	486	449

Values represent total number of students. We did not have complete demographic data for all students. Students participating in the study could select more than one ethnicity and more than one gender identity.

### Measures

#### Perceptions of other students

We measured students’ perceptions of women’s and men’s abilities in life and physical sciences by asking participants to identify fellow students in their participating class(es) using a protocol informed by Grunspan et al. [13]. Based on the course and lab section in which they were enrolled, participants were given a list of other students in the course/lab by first name and included a last initial when there was more than one person with the same first name. Each name had a check-box next to it, and participants were asked: “Do you study with any other students in your [x] course (e.g., for homework or exams)?”; Are there any students in your [x] class that you are more likely to go to if you need help in the class (e.g., if you have questions or need study notes)?”; “Thinking about your [x] course, do any students stand out as particularly knowledgeable?” and “Thinking about your [x] course, who would you consider to be the best student(s) in the class?” In cases where there was a laboratory section, the words “course” or “class” were replaced with “lab section.” Participants were also provided with the following set of directions after each question: “If you do not know a student’s name, please check "other,” and write a short description of that person (e.g., hair color, gender, or other distinguishing characteristics).” For the question about the best students in the course, the following was included: “If you consider yourself to be one of the best students, please write ‘myself.’ Please list at least 2 students. You may list up to 5 students.” For this question, participants were asked to rank the best students by providing a 1–5 next to each name. There were two “other” options provided in each class list.

Our choice of methodology (i.e., having students list up to five other students in their course) is intentionally similar to the methods of Grunspan et al. [13], who allowed students to list an unlimited number of peers when they collected data but focused on the top five students in a class in their analysis. The purpose of selecting other students in the course was to determine whether participants were more likely to think of women or men as those they studied with, went to help for, or perceived to be the best students in the class. Thus, we calculated the proportion of women chosen for each category, out of the total number of students each participant selected in that category. This created a measure ranging from 0 to 1 with scale values that were meaningfully equidistant from each other. If no students (women or men) were selected for a category, the proportion of women selected was computed as a missing value.

#### Gender of participants and classmates

Each student on the course roster was coded by the gender they selected on the survey. The survey included several gender options, including a space for students to fill in their own gender identity. If a student did not complete the survey or provide their gender on the survey, we used their gender as indicated via the university registrar. In cases where participants did not select a named classmate on the list, but instead checked “other,” the classmate was coded as a woman or man based on the pronouns used in the description by the participant (e.g., “she sits in the very front,” or “he has black hair”). If the description did not specify the classmate’s gender, the answer was not counted.

#### Academic performance

At the completion of the semester, course letter grades and overall university-wide grade point averages (GPAs) were obtained for each student in the participating courses through the university registrar’s office, regardless of whether they participated in the survey. Course letter grades were converted to numeric scores on a traditional 4.0 GPA scale (A+ or A = 4.0, A- = 3.67, B+ = 3.33, B = 3.0, B- = 2.67, C+ = 2.33, C or C- = 2.0, D = 1.0, F = 0). Grades of W (withdraw) or I (incomplete) were calculated as missing values. University GPAs were collected on a 4.0 scale. Students who were completing their first semester of college at the institution where the study took place did not have university GPA data available (49% of the total sample). Finally, analyses examining top performing students in each course differentiated between those who earned a course letter grade of A or A+ versus any other grade.

## Results

### Preliminary analyses

We conducted preliminary analyses to verify if there were any major factors that influenced whether students participated in the study or differed on our variables of interest. Missing data analysis indicated that some demographic variables significantly predicted whether or not a student participated in the survey: students who identified as STEM majors (83% of the survey participants), students who identified as White, and those taking physical science courses were more likely to participate than non-STEM majors, racial-minority students, and those taking life science courses (all *p*’s < .05). In addition, students who participated in the survey had statistically higher standardized course grades, *F*(1, 2724) = 11.41, *p* < .01, *M*_*survey*_ = 0.08, *M*_*non-survey*_ = -0.06, and overall university GPAs, *F*(1, 1456) = 43.23, *p* < .001, *M*_*survey*_ = 3.13, *M*_*non-survey*_ = 2.91, than students who did not participate. Moreover, STEM majors had higher grades than non-STEM majors, but this was confounded with being an upper level (e.g., fourth year) versus a lower level (e.g., first year) student. Therefore, we cannot separate the effects of being a STEM major on grades from level in college, age, or potentially other confounding factors. No other results based on demographic data were statistically significant.

Because of the nested nature of the students (individual level data) within nine classrooms (second-level data), we calculated interclass correlation coefficients to determine the variance explained by nesting within classrooms. Average classroom size was 318 students per class. Classroom accounted for 27% of the variance in the proportion of women selected as the best in the class (ICC *r* = .2738) and 8% of the variance in grade distributions (ICC *r* = .079). Thus, we used multilevel modeling with Maximum Likelihood estimation in the following analyses to account for non-random variability explained by nested data.

### Academic performance

We conducted a linear multilevel model with classroom as the level-2 nesting variable, student gender (women vs. men) and course type (life vs. physical science) as the predictor variables, and course grade as the outcome variable. Results indicated that gender was a significant predictor of course grade, *F* (1, 2794.9) = 4.22, *p* < .05, with women having slightly higher grades (*M* = 3.01, *SE* = 0.12) in both life and physical science courses than men (*M* = 2.90, *SE* = 0.11). There were no statistical differences found in course grade based on course type and the interaction between course type and gender was not significant.

We also examined whether women or men would perform as the top students in the class (as defined by earning an A or A+) as a function of course type, controlling for nesting within classrooms. We used a generalized linear multilevel model with whether or not the student earned a top grade as a binary logistic outcome measure. Results indicated a marginal main effect of gender, with women being about 1.5 times more likely than men to earn an A or A+, *B* = 0.393, *SE* = 0.205, *p* = .056, *OR* = 1.48, *95% CI* [0.99–2.21]. There were no effects of course type on likelihood of earning a top grade, nor a significant interaction.

Finally, we examined whether gender predicted university GPA as an outcome variable. Because of the likelihood that students’ GPAs will change over the course of their college career, we included student level (1^st^, 2^nd^, 3^rd^, or 4^th^ +) as a second predictor variable using univariate ANOVA. Results showed a significant main effect of student gender, with women (*M* = 3.05) having significantly higher university GPAs than men (*M* = 2.93) overall, *F* (1, 1448) = 4.06, *p* < .05, *η*_*p*_^*2*^ = .003. We also conducted four linear multilevel models using the proportion of women in each class who earned a top grade as a covariate instead of the total number of women in the class. All effects remained in the same direction and were either similar to or slightly larger than those reported above.

### Perceptions of other students

To examine perceptions of other students, we conducted four separate linear multilevel models like those described above (using participant gender and course type as predictor variables and individual classroom as the level-2 nesting variable) to examine the proportion of women vs. men selected as those the participants: 1) chose to study with; 2) chose to ask for help; 3) found most knowledgeable, and 4) identified as the best students in the class. In all cases, we controlled for the total number of women (versus men) in the class. Results for all four multilevel models indicated a main effect of participant gender and a main effect of course type, with no interactions, as specified below.

Women (*M* = 59.4%) were more likely than men (*M* = 32.9%) to select a higher proportion of women students with whom they chose to study, *F* (1, 541) = 61.31, *p* < .001, and women (*M* = 52.4%) were more likely than men (*M* = 34.3%) to select a higher proportion of women students from whom they sought help, *F* (1, 663) = 30.86, *p* < .001. Similarly, women (*M* = 49.7%) selected a higher proportion of women to men as the most knowledgeable in their class compared to men (*M* = 33.7%), *F* (1, 553) = 18.71, *p* < .001 and were more likely (*M* = 49.5%) to identify a higher proportion of women as the best students in the class compared to men (*M* = 32.4%), *F* (1, 791.88) = 50.75, *p* < .001.

**Fig 1:**
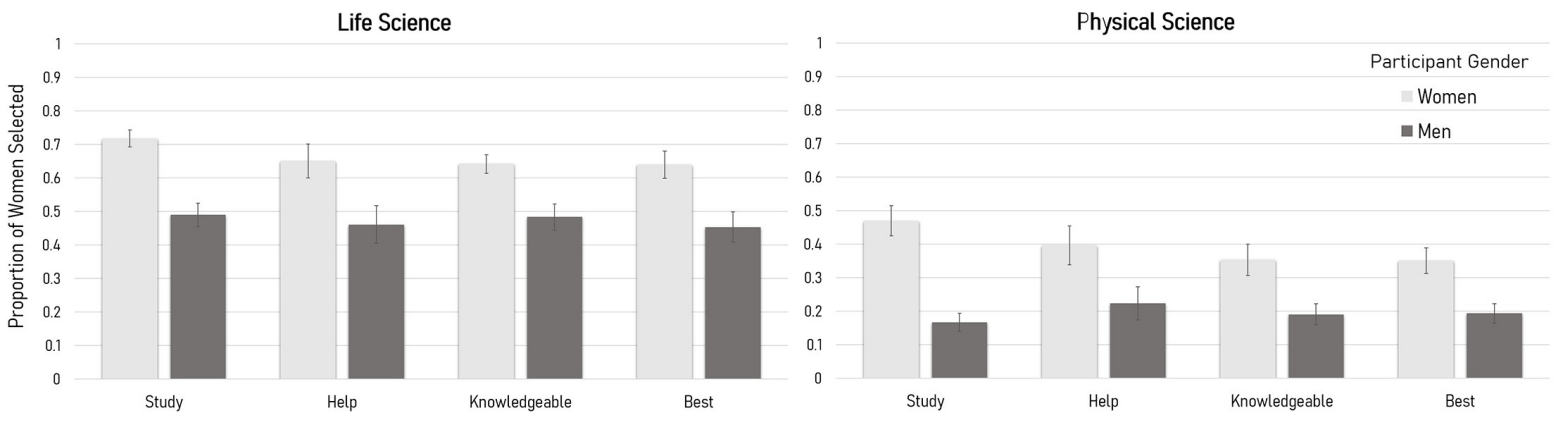
Average proportion of women selected for each category by participating women and men who were students in physical and life science courses. Confidence intervals that include 0.5 indicate an equal proportion of women to men selected in each category. Values higher than 0.5 indicate that a higher proportion of women to men were selected. Values lower than 0.5 indicate that a higher proportion of men to women were selected.

Four significant main effects of course type revealed that participants in life science courses (*M* = 60.4%) were more likely than participants in physical science courses (*M* = 31.9%) to select a higher proportion of women students with whom they study, *F* (1, 541) = 71.00, *p* < .001, and participants in life science courses (*M* = 55.6%) were also more likely than participants in physical science courses (*M* = 31.1%) to select a higher proportion of women students from whom they sought help, *F* (1, 3.88) = 12.85, *p* < .05. Following the same pattern, participants in life science courses (*M* = 56.3%) were more likely than participants in physical science courses (*M* = 27.2%) to indicate that a higher proportion of women were the most knowledgeable, *F* (1, 553) = 61.88, *p* < .001, as well as more likely (*M* = 54.7%) than participants in physical science courses (*M* = 27.3%) to select a higher proportion of women as the best in the class, *F* (1, 1.59) = 45.83, *p* < .05 (Fig 1). We also found a significant effect of student level, *F* (3, 1448) = 17.54, *p* < .001, *η*_*p*_^*2*^ = .035 and a significant interaction between level and gender, *F* (3, 1448) = 9.36, *p* < .001, *η*_*p*_^*2*^ = .019. Post-hoc tests using the Bonferroni correction examining the interaction effect indicate that GPAs were significantly lower for 1^st^ year students than any other level, with GPAs among 2^nd^, 3^rd^, and 4^th^+ students not varying significantly. The post-hoc test of the interaction showed men in their first year of college have significantly higher university-wide GPAs than women in their first year, while women in their second and third years have higher GPAs than men in those years, and there is no statistical difference between the GPAs of senior-level women and men.

## Discussion

This is the first study of which we are aware to document that women are outperforming men in both physical and life science courses, while men simultaneously continue to be perceived as equal or better students. We found that 1) in both life science and physical science courses, women had statistically higher course grades than men, higher university-wide GPAs than men, and were 1.5 times more likely to earn an A or A+ than men, and yet 2) both men and women identified women as students they study with, seek help from, find knowledgeable, and perceive as the best in the class at lower proportions than the actual success rates of women in their classes. Although men consistently underestimated women classmates across both life and science fields more often than women do, there is also a stark contrast between the perceptions of women’s abilities in the physical sciences compared to the life sciences. In the physical sciences, both women and men were disproportionately less likely to select women than men as those they seek help from, find knowledgeable, and perceive as the best in the class. In contrast, in the life sciences, men were equally likely to identify a woman or a man in all categories while women identified women and men equally as the best in the class and were more likely to identify women than men for the other categories. However, since women outperformed men across both life and physical sciences, picking an equal number of men and women is still underestimating the performance of women in the class.

The results of this study demonstrate that gender bias persists in undergraduate STEM classrooms that incorporate student-to-student interactions even when women outperform men, and even in areas where women now outnumber men (life sciences). Although we did not find evidence of gender bias specifically affecting women’s performance in their courses, others have found a relationship between gender and performance during group work activities in STEM courses. Studies of physical science courses found that performance is higher for women when they are placed in single-gender groups compared to their counterparts in mixed-gender groups; men’s performance, however, was not reportedly affected by their group members’ gender [27,28]. Therefore, our study is relevant at a time when group work is being encouraged as an instructional strategy, in spite of the different types of interaction (e.g., think-pair-share discussions, laboratory activities) students experienced in our study. A recent meta-analysis of 225 studies found that STEM courses in which active learning strategies were employed, student performance on exams increased [29]. While increased opportunities for collaborative work or interaction may be important for increasing performance of women in physical science courses (e.g., [30]), another study [31] found that interactive educational environments are not sufficient to reduce the gender gap and that other factors play a role. Some scholars claim that better understanding women’s experiences during undergraduate group work is important in maintaining women in STEM fields because women’s performance increases as their numbers increase in small groups [32]. It is clear that more research on classroom interactions and performance is needed. Our study is particularly relevant for introductory STEM courses, which often have laboratory sections in which students interact with one another in groups. These results also support prior research that shows that women are less likely than men to engage in gender bias [33] and that gender bias is less prevalent in disciplines in which women have similar or greater representation than men compared to those in which they are underrepresented [34]. Yet, importantly, gender bias still exists among women themselves and in disciplines in which women are equally represented.

The finding that women continue to experience bias even though they outperform men may not be particularly surprising given the abundance of social science research on stereotype information processing. Once stereotypes are formed, information that is inconsistent with one’s stereotype or worldview is often discounted, forgotten [35], or processed as a subtype that does not challenge the overarching stereotype [36]. For example, women’s generous gains in academic performance can be explained in system-justifying ways [37], such as the recent assertion that boys have become academically lazy while girls work hard (e.g., see [38]), reinforcing the perception that men have innate ability and talent, particularly in STEM, while women must work hard to overcome their natural ineptitude to achieve results [39]. Skeptics of women’s achievements point to the variability hypothesis, which suggests that men’s average achievement may be similar to women’s, but their greater variability accounts for more men found among the upper echelons of brilliance [40]. In line with recent research [41], this hypothesis was not supported in the current study, showing that women were more likely than men to earn top grades across both types of science courses. The wide-spread gender bias favoring men’s greater earnings, promotions, and overall representation in STEM fields cannot be explained in this case by their greater variability in college performance or their greater likelihood of being elite students.

While it may appear, in our study, that gender bias did not prevent women from performing equal to or better than their peers who were men, these results still have important implications for gender diversity in STEM. The endorsement of gender bias can lead aspiring women to decrease their interest in STEM, their self-efficacy, and their performance both individually and in teams, and ultimately lead these women to pursue other disciplines [42,16]. Research demonstrates that more subtle factors, such as whether women often attend group study sessions, are significant predictors of completing a STEM degree [43]. Thus, our measures of whether women were sought by fellow students to study with or receive help might have implications on their graduation rates in STEM.

These impacts may be exacerbated for women of color [44]. Black women in STEM can face multiple forms of bias and discrimination, including an intersectional effect of being a Black woman that White women and Black men do not experience [45]. However, some research points to cultural buffers that Black women might experience due to less internalization of gender-role stereotypes among African Americans [46]. Asian women also face conflicts between gender and racial stereotypes regarding abilities in STEM [47], and their experiences are often discounted because of assumptions that Asians excel in STEM [48]. We did not have a large enough sample of women of color to statistically identify intersectional effects and did not have information about sexual orientation about participants. Thus, our results are not representative of all women’s and other gender-minorities’ experiences but shed some light on the continuation of bias towards all women, regardless of their academic performance.

### Limitations

One limitation to the method of gathering students’ gender was that we may not have accurately recorded all students’ gender identity. Although our survey was designed to allow participants to self-identify their own gender, when they referred to classmates, we recorded how they perceived the gender of their classmates. For students who did not self-identify gender or referred to classmates as “other,” we relied on university records, which, at the time of the study, was limited to a binary gender identification. We acknowledge that this method for measuring students’ gender is not always accurate (e.g., students may change their gender identity without updating their information with the university registrar, which currently allows “x gender,” or participants may misperceive another student’s gender identity) and may have had some effect on the outcomes measured.

A second potential limitation to the study was the non-representative sample of survey participants compared to the overall demographics of the courses themselves. Missing data analysis revealed that students who participated in the survey were more likely to be STEM majors, White students, physical science students, and students with higher class grades and GPAs. This may have skewed our results toward the perceptions of higher-achieving, STEM- and physical science-focused White students, who, based on the previously reported literature, may be more likely to harbor gender STEM stereotypes than other students. Additionally, we cannot say for certain that participants were accurate or inaccurate in judging the best students in their class. For instance, in classes where more than 5 women and 5 men earned A’s, participants could have correctly identified 5 of the top students in the class by selecting all men. Participants could also have incorrectly identified some or all of the top students in the class, which could include both women and men. However, we argue that regardless of accuracy, the choice to select more men than women as the top students on average in classes where women outnumber men as the best students represents a form of gender bias. If no bias were present, then we would expect that either a) participants’ selections would average out to an equal number of women and men as the best students (either accurately or inaccurately), or b) participants would select the same proportion of women to men as the proportion of women to men in the class overall. Given that we largely find discrepancies between these possibilities and the proportion of women to men that participants actually selected, we conclude that regardless of whether participants were accurate, they still exhibited gender bias in their selections.

Third, the low overall representation of students and particularly women of color in both the participant sample and the courses themselves limits our ability to reflect the specific perceptions of and about these students in undergraduate science fields. Furthermore, because the small number of minority students was spread across many lecture classes and labs, and because participants only rated the other students within their own classes, we did not have enough statistical power to conduct intersectional analyses (average number of minority women within a course/lab was *n* = 2). An abundance of important work on intersectional experiences of bias indicates that it is important to differentiate the experiences of women of color, transgender and non- binary people, women from lower socio-economic backgrounds, and other social categories that are used to marginalize people. Although we cannot account for the differential bias that very likely affects women of color in our study and acknowledge that women of color are not accurately represented in these results, we caution readers that our results largely reflect the experiences of White cisgender women and that these results should be interpreted as such.

### Future directions and conclusions

How can this problem be overcome? Intergroup contact and cooperation are foundational elements to reducing stereotyping, prejudice, and discrimination [49]. As such, group work is often promoted as a tool to improve science education [50] and is associated with improved student understanding and performance [51]. Some studies have demonstrated that women prefer or appreciate cooperative group work [52]; however, other studies highlight the diminished benefits of group work for women [53], such as women being less likely to have their suggestions adopted or be given credit for important work [54]. When students select their own groups, they report feeling at greater ease and are more trusting of one another, although performance within self-selected and instructor-selected groups does not necessarily differ [50].

Interpersonal backlash toward women who succeed in stereotypically masculine fields, such as STEM, can lead women to downplay their accomplishments and maintain cultural stereotypes during interactions with peers [55]. We assert that women’s performance and accomplishments in STEM need to be publicly recognized to address misperceptions that women are less capable, skilled, or have less expertise. Moreover, being aware of other women’s success has the potential to retain women in STEM who might instead pursue other fields of study. Representation of marginalized groups in undergraduate STEM classrooms is correlated with marginalized students’ increases in self-efficacy, effort, and feelings of connectedness to the subject [8] and stereotypes can change over time, given enough counter-stereotypic examples [56,57,36]. Hence, for institutions committed to diversifying their undergraduate population, the results of this study are relevant and timely.

Women are excelling in STEM, and yet they still contend with biased perceptions of their abilities. Our study demonstrates that women can still be subject to gender bias *even when* they outperform and outnumber their male counterparts in undergraduate STEM classrooms. Moreover, our study shows this effect across both physical and life science courses and lower and upper level courses. We argue that theory and research on gender bias and performance must address why gender bias continues to be exhibited in undergraduate classrooms despite women excelling. Institutions of higher education should have a vested interest in breaking down biased perceptions of women and other marginalized groups in their STEM classrooms, especially if they are committed to diversifying STEM [58] and supporting high-quality science through gender diverse research teams [59]. Therefore, we implore university educators to address the problem of gender bias, both through interventions and explicit discussions with students, rather than the problem of gender performance disparity or representation.
